# Perspectives of nurses regarding pain assessment and management during routine infant vaccination in Ghana

**DOI:** 10.1002/nop2.1772

**Published:** 2023-05-23

**Authors:** Emma Annan, Tendani S. Ramukumba, Bonnie J. Stevens

**Affiliations:** ^1^ University of Ghana Accra Ghana; ^2^ Tshwane University of Technology Pretoria South Africa; ^3^ Lawrence S Bloomberg Faculty of Nursing University of Toronto Toronto Ontario Canada

## Abstract

**Aim:**

To explore the perspectives of nurses regarding pain and its management during routine infant vaccination at the Child Welfare Clinics in Ghana.

**Design:**

Qualitative descriptive design.

**Methods:**

Qualitative in depth, in‐person interviews using a semistructured interview guide were conducted with 19 Registered Nurses who were were purposively sampled from three selected Child Welfare Clinics in hospitals in the Greater Accra Region of Ghana, The Tesch cotent analysis procedure was followed for the analysis of interview data.

**Results:**

Nurses were aware that the injections they give infants are painful. They described how infants exhibit certain behaviours to express pain. Although nurses support infant pain management during vaccination, they rarely use evidenced‐based pain interventions.

## 1 INTRODUCTION

Since its introduction, vaccination has drastically reduced vaccine‐preventable diseases and deaths in sub‐Saharan African countries such as Ghana (World Health Organization, 2015). Despite its benefits, vaccine injections are the most common painful needle procedure in the first year of life (Göl & Altuğ Özsoy, 2017; Mudundi et al., 2020). Unmanaged pain during vaccination can lead to future healthcare avoidance, fear of needles and poor vaccination compliance, leading to vaccine‐preventable disease outbreaks (Taddio, 2021).

## 2 BACKGROUND

There are several evidence‐based strategies to reduce pain during vaccination of infants, such as such as breastfeeding, sweet solutions, proper position during vaccination and caregiver presence (Taddio, McMurtry et al., 2015, Taddio, Shah et al., 2015; World Health Organization, 2015). Evidence from high‐income countries shows that nurses administering vaccines to infants do not adequately manage their pain and expose them to unnecessary pain (Harrison et al., 2018). This gap between knowledge and practice has also been noted in studies in low‐ to middle‐income countries such as Benin (Adedemy et al., 2020).

There is a paucity of data from the nurses' perspectives on infant pain and management during vaccination in Ghana. The role of nurses in managing pain in infants during routine vaccination plays a crucial role in preventing both the short‐ and long‐term consequences of pain. Therefore, nurses' perspectives on infant pain and its management during routine vaccination at the Child Welfare Clinics (CWC) in Ghana were explored.

## 3 METHODS

### 3.1 Design

The study was qualitative and descriptive using the content analysis method (Sandelowski, 2000).

### 3.2 Participant selection and setting

The study was conducted in three selected hospitals in the Greater Accra Region of Ghana. Hospitals included a regional hospital, a district hospital and a children's hospital. The purposive sampling technique was used to recruit 19 Registered Nurses who worked as nurses in the CWC. Inclusion criteria were at least 3 months of work experience at the CWC. The number of nurses participating in the study was determined by saturation at which no new information was generated (Fusch & Ness, 2015).

### 3.3 Data collection

The method of data collection was a semistructured individual face‐to‐face interview. The interview guide was designed to explore nurses' experiences with infant pain assessment and current pain management practices during vaccine injections. The interview guide consisted of two sections: The first section included demographic information about nurses, and the second section consisted of open‐ended questions on beliefs, perceptions and experiences about infant vaccination pain, assessment and management during vaccination.

After obtaining approval from hospital administrators and Deputy Directors of Nursing Services at the selected sites, the lead author contacted the heads of units at the CWCs. With the support of the unit heads, nurses who met the criteria were invited to be interviewed. The interviews were conducted at the convenience of the participants in the nurses' room. Each interview lasted for 45–60 min. Interviews were audiotaped with the permission of the participants. Data were collected until data saturation was reached. This is the point where no new data was found.

### 3.4 Data analysis

Data analysis was carried out using Tesch's guidelines for content analysis. This approach categorizes verbatim transcriptions into themes for analysis (Creswell & Creswell, 2018). The data were transcribed verbatim. The transcriptions were carefully read several times to gain understanding, and the thoughts and ideas that came up were written down. Notes of ideas from the data with similar topics were arranged into groups. The topics were then abbreviated as codes, and codes were written next to the appropriate text segments, using the most descriptive wording for the topics, and grouping topics related to each other into categories. The data belonging to each category were brought together in one place; the categories were grouped into themes described and supported by subcategories with their quotes. A preliminary analysis was performed. The organization of the data was observed to check for new categories or codes emerging. The biographical data were analysed quantitatively.

### 3.5 Trustworthiness

The criteria of credibility, transferability, reliability and confirmability were evaluated to determine trustworthiness in the study (Lincoln & Guba, 1985). The lead author was reflective during the study process, especially during data collection, and the first two interviews were coded separately by the lead author and an independent coder. Differences and similarities were recognized and corrected. Dependability was ensured by a detailed description of the research settings and the study. The lead author described the circumstances and participants to establish reliability. An audit trail of interviews, transcripts, and report drafts ensured data confirmability. Participants' replies were recorded verbatim, and themes were substantiated by direct quotes.

### 3.6 Ethical considerations

Research Ethics Committee approval ‘REDACTED’. At the selected sites, the permission of the administrators and the Deputy Directors of Nursing Service was obtained. All nurses were informed of the purpose of the study and signed informed consent forms. They were told that participation was voluntary, and the confidentiality of the information was assured. All interviews were audiotaped with the consent of the participant.

## 4 RESULTS

The data were collected in 2019 during working hours between 9:00 a.m. and 2:00 p.m. A total of 19 nurses participated in the study.

Most of the nurses were in the age range of 30–39 years. All but one of the 19 nurses were females, with 16 (84.2%) having certificate educational qualifications in nursing. Over a quarter of the interviewed nurses were community health nurses (26.3%, *n* = 5) or senior community health nurses (26.3%, *n* = 5). Table 1 provides a detail of the participants' characteristics.

**TABLE 1:** Demographic characteristics of nurses (*n* = 19).

Factor	Sites			Total
	Site 1	Site 2	Site 3	
Number of participants	8	5	6	19
Age (years), median (IQR)	37 (32.5, 46.5)	30 (26, 32)	34 (30, 38)	34 (30, 39)
*Age group (years)*				
<30	1 (12.5)	2 (40.0)	0 (0.0)	3 (15.8)
30–39	4 (50.0)	3 (60.0)	5 (83.3)	12 (63.2)
40–50	3 (37.5)	0 (0.0)	1 (16.7)	4 (21.1)
*Gender*				
Female	8 (100.0)	5 (100.0)	5 (83.3)	18 (94.7)
Male	0 (0.0)	0 (0.0)	1 (16.7)	1 (5.3)
*Educational qualification in nursing*				
Advance diploma	0 (0.0)	0 (0.0)	1 (16.7)	1 (5.3)
Certificate	7 (87.5)	5 (100.0)	4 (66.7)	16 (84.2)
Degree	0 (0.0)	0 (0.0)	1 (16.7)	1 (5.3)
Diploma	1 (12.5)	0 (0.0)	0 (0.0)	1 (5.3)
*Duration of service at CWC*				
<5	0 (0.0)	3 (60.0)	2 (33.3)	5 (26.3)
5–9	3 (37.5)	1 (20.0)	3 (50.0)	7 (36.8)
10+	5 (62.5)	1 (20.0)	1 (16.7)	7 (36.8)
*Current rank in nursing*				
Community health nurse	1 (12.5)	3 (60.0)	1 (16.7)	5 (26.3)
Principal community health nurse	2 (25.0)	1 (20.0)	1 (16.7)	4 (21.1)
Senior community health nurse	2 (25.0)	1 (20.0)	2 (33.3)	5 (26.3)
Senior nursing officer	0 (0.0)	0 (0.0)	2 (33.3)	2 (10.5)
Staff nurse	1 (12.5)	0 (0.0)	0 (0.0)	1 (5.3)
Superintendent community health nurse	2 (25.0)	0 (0.0)	0 (0.0)	2 (10.5)

Two principal themes were identified: (a) Vaccinations are painful for infants and (b) The current pain assessment and management practices by nurses in Ghana during vaccination are inadequate. The first theme consisted of three subthemes namely (i) infants can experience pain during vaccination, (ii) infants express pain in different ways during vaccination, and (iii) nurses' beliefs about factors that make vaccination painful. The second theme consists of four subthemes namely (i) nurses do not assess pain using a validated tool, (ii) nurses do rate pain informally, (iii) nurses are supportive of managing infant pain, and (iv) current pain management practices are limited.

### 4.1 Theme 1: Vaccinations are painful for infants

Nurses reported that infants feel pain during vaccination and may express pain in a variety of ways, as well as their beliefs about factors that make vaccination painful.

#### 4.1.1 Infants can experience pain during vaccination

All nurses (*n* = 19) were very aware that infants can and do experience pain during vaccination. Their demonstration of awareness focused on their observation that infants showed signs of pain when injected.

When they are on their mother's lap, they do not cry but the moment you inject them, they start crying; that means that they felt the painPARTICIPANT 3 SITE 1.

#### 4.1.2 Infants express pain in different ways during vaccination

All participants (*n* = 19) reported that infants expressed pain through behaviour, including vocal expressions, negative facial expressions and body movements during vaccination.

- Vocal expression

All nurses (*n* = 19) reported that vocal expressions such as crying or screaming were the most common signs of infant pain during vaccination.

Immediately you inject them they tremble and start cryingPARTICIPANT 5 SITE 1.

ii Facial expression

Most nurses (*n* = 14) reported that all infants exhibited pain in their facial expressions during immunization, characterized in most cases by frowns or grimaces, tightly closed eyes and opening their mouths wide when they cry.

Some of them frown their faces or grimace immediately you injected themPARTICIPANT 3 SITE 3.

When they cry, they open their mouths wide and close their eyes tightly to tell you they are in painPARTICIPANT 1 SITE 1.

iii Body movements

Some nurses (*n* = 7) reported that infants usually cannot keep still when they are in pain. Limb movement is one of the ways they communicate the pain they are going through. They described how some infants withdraw their limbs in response to pain during vaccination.

They shared how aggressive and restless some infants become when expressing pain. They do this by violently throwing their limbs and shaking their heads.

As soon as you inject them, they withdraw their limbs to express pain, and when you touch the injection site, they withdraw that limb, and this tells you that they are in painPARTICIPANT 1 SITE 3.

They show aggression by shaking their heads and throwing their limbs about when you administer the vaccine injectionsPARTICIPANT 6 SITE 1.

#### 4.1.3 Nurses' beliefs about factors that make vaccination painful

All nurses (*n* = 19) noted that the number of injections and both tissue damage and deposition of vaccine components in the skin cause infants to feel pain during vaccination.

- Number of injections

Most nurses (*n* = 14) firmly believed that multiple injections per visit were the cause of the pain. They mentioned that infants could have up to three injections per visit, which they felt was too painful, and they complained about the frequency. A nurse feared even more painful vaccines could be introduced due to emerging diseases.

My biggest concern is the fact that every time new diseases emerge, new vaccines are added to the existing ones. As before, we only gave pentavalent vaccine on the left thigh. Now they've added IPV, so for example at 14 weeks we give the babies three injections and that causes excruciating pain for the babiesPARTICIPANT 6 SITE 3.

The mothers complain about the multiple injections because of the pain their babies experience, they are usually not happy when their babies are due for multiple injectionsPARTICIPANT 2 SITE 2.

ii Both tissue damage and deposition of vaccine constituents into the skin.

A few of the participants (*n* = 5) believed the cause of pain the infants feel during vaccination is the result of both tissue damage and deposition of vaccine constituents into the skin.

The needle that is going through the skin gives pain and pushing the vaccine constituents into the skin gives pain, so both together are very painful for the childPARTICIPANT 4 SITE 1.

### 4.2 Theme 2: Current pain assessment and management practices by nurses in Ghana are inadequate

The findings indicated that all the nurses did not assess pain during vaccination. Evidence‐based pain interventions were rarely used, even though they supported pain management during infant vaccination.

#### 4.2.1 Nurses do not assess pain using a validated tool

Nurses did not assess pain using a validated assessment tool because they had not received any formal education on pain assessment during infant vaccination. Others attributed this to their lack of time and lack of knowledge in managing pain.

- No formal assessment

All nurses (*n* = 19) reported that no formal assessment of the infant's pain was performed during vaccination. Most participants were unaware of their role in pain assessment, while others did not even know how to assess pain.

I do not know that I have to assess pain during immunization, I think my duty is to immunize babiesSITE 3 PARTICIPANT 5.

ii Lack of time

Almost half of the participants (*n* = 9) explained that their reasons for not doing formal assessments were due to the lack of time during vaccination. The lack of time usually arose from the high number of infants they (nurses) must serve, making it difficult for them to assess pain.

There is no time to assess vaccination pain even if we are educated in vaccination pain assessmentPARTICIPANT 4 SITE 3.

The mothers who bring their infants to vaccinated are in a hurry. So, if you spend time on one baby assessing the pain, they will throw insults at you (laughs) so the time will be a challenge even if we knew how to conduct the assessmentPARTICIPANT 5 SITE 3.

iii Lack of knowledge

All of the nurses (*n* = 19) said they lacked knowledge on how to assess pain.

{Hesitant} no we do not assess pain during vaccination, I am not sure how to assess pain during vaccinationPARTICIPANT 2 SITE 3.

#### 4.2.2 Nurses do rate pain informally

Nurses perceive pain intensity without using a formal assessment tool. Responses from nurses indicate that most nurses (*n* = 11) perceive the intensity of pain that infants experience as moderate, even though they did not use any formal way of assessing.

- Nurses do rate pain as moderate

They (nurses) usually attributed this rating to the duration of infants' cries after the vaccination and the type of procedure. To them, vaccination is a minor procedure that will not bring about severe pain.

It is moderate because they cry briefly and then stopPARTICIPANT 1 SITE 3.

If I have to rate the pain, I will rate it as moderate because it is a minor procedurePARTICIPANT 2 SITE 2.

ii Nurses do rate pain as high

Some of the nurses (*n* = 8) viewed infant pain to be of high intensity. This is attributed to several injections administered to the infant at one time, causing much pain.

Due to the multiple injections, I rate it as high, three injections per visit lead to severe painPARTICIPANT 7 SITE 1.

#### 4.2.3 Nurses are supportive of managing infant pain

All the participants (*n* = 19) agreed that they supported managing infant pain during vaccination. They explained that since pain is a natural phenomenon for humans, its management is necessary as it helps to reduce the pain the infant feels.

Yes, it should be managed so that their pain will be lessened, and crying will stop. The pain increases the temperature of the babies and sometimes they have to be hospitalized which will incur a cost for the parentsPARTICIPANT 2 SITE 1.

#### 4.2.4 Current pain management practices are limited

Most nurses did not use evidence‐based interventions to manage infant pain during vaccination.

##### Nurses do not use evidence‐based interventions to manage pain

Most nurses (*n* = 15) did not use evidence‐based interventions to manage infant pain. Rather, they (nurses) usually advised parents to use paracetamol syrup, shea butter and apologetic words to reduce pain.

- The use of paracetamol syrup

Nurses (*n* = 7) told mothers at the site to give paracetamol syrup to their babies after the injections.

Mm (pause) when they come here, we mostly tell them to give them some paracetamol when they get home, paracetamol is mostly for their feverPARTICIPANT 1 SITE 3.

ii The use of shea butter

Some nurses (*n* = 3) advised the mothers to use shea at the site of the injection to help reduce pain.

Sometimes we tell the mothers that if their babies develop an abscess at the injection site, they should put an ice pack on the injection site. After that, they can smear shea butter to relieve the pain of the injectionPARTICIPANT 6 SITE 1.

iii Apologetic words

Some of the nurses (*n* = 5) spoke about how apologies were rendered to infants after injection. These apologies (especially words like sorry) are said by the mothers and/or nurses to manage the pain. Apologies are thought to be effective in managing pain as it calms the infant down.

Sometimes when we inject the babies, we tell them ‘Sorry, sorry’ or try to pamper themPARTICIPANT 1 SITE 1.

###### 4.2.4.1 No interventions

Some of the nurses (*n* = 4) explained that they did nothing to reduce infants' pain during vaccination. Rather, they left the pain management to the mothers. Mothers will have to use their knowledge to manage the pain of their infants, be it right or wrong.

Currently, just after the injections, we do not do anything for them. Some of the mothers have older children so they know what to do. They have paracetamol syrup in their bags so immediately you inject the babies they administer it. Some mothers even administer it at home before coming herePARTICIPANT 4 SITE 1.

We don't do anything here, the only thing we do is to put the cotton at the site because we have opened the skin. So, we put the cotton wool there to stop the bleeding after the injectionPARTICIPANT 7 SITE 1.

## 5 DISCUSSION

Vaccine injections are the most common cause of iatrogenic pain in infancy (Hall et al., 2020). The nurses in this study expressed awareness of pain in infants during vaccination, a finding consistent with research reports that nurses and physicians believe vaccination causes pain but pain management during vaccination has been underutilized (Porter et al., 1997; Taddio et al., 2009). This contradicts the earlier beliefs of clinicians who once believed that infants did not experience pain because they are too immature to feel pain (Stevens et al., 2021). Although the existing literature is replete with evidence that infants experience pain, some nurses in LMIC still believe that infants do not experience pain. More studies are needed to elucidate pain in infants. For example, in a study on pain management in newborns in Rwanda, most nurses and midwives (80.3%) reported that newborns do not experience pain (Muteteli et al., 2019).

Like previous studies (Adedemy et al., 2020), nurses in this study indicated that infants exhibited pain via different behavioural expressions through vocal expression (crying or screaming), facial expression (frowning or closing eyes tightly) and body movements (withdrawing or throwing limbs, aggression by shaking the head). These findings are consistent with Taddio's Modified Behavioural Pain Scale (MBPS), which identified facial expression, crying and body movements as parameters of pain during infant vaccination (Taddio et al., 1995). The ability of nurses to recognize the pain responses of infants in their care is the most fundamental part of good pain management.

Although all nurses in this study supported the management of infant pain during vaccination (Chan et al., 2013), they typically advised parents to use paracetamol syrup, shea butter compresses and apologetic words to relieve pain. These interventions are not evidence‐based interventions. Previous studies have shown that health workers use these less effective pain management interventions while vaccinating infants (Adedemy et al., 2020; Russell & Harrison, 2015; Saul, 2017). Studies have shown that pain relief measures are not always taken during vaccination of infants (Harrison et al., 2014). This is supported by the results of this study, in which nurses did not use WHO‐recommended evidence‐based pain management measures in infants during vaccination. According to WHO recommendations, nurses administering vaccines to infants should use strategies such as breastfeeding, administration of sweet solutions, proper positioning, caregiver presence and distraction to reduce pain. These interventions are inexpensive and do not require much time to implement (10.1016/j.vaccine.2015.11.005).

Mothers have expressed concern about the pain associated with vaccinations in infants, which would lead to vaccination delays. Applying these evidence‐based strategies during infant vaccination has been shown to be an effective strategy to overcome parental vaccination reluctance and ultimately improve the quality of infant care (World Health Organization, 2015).

Some of the nurses reported that they did nothing to relieve the infants' pain during vaccination. Rather, they left pain management to the mothers. There is evidence that parents may not receive the support of healthcare providers when it comes to pain interventions. (Parvez et al., 2010). McNair et al. (2017) showed that mothers often use nonpharmacological strategies such as diverting and acting calm to ease pain during infant vaccination. Mothers use ineffective methods as reassurance intuitively, but not because nurses tell them to (Taddio et al., 2012). Nurses in several geographic locations around the world have been shown not to use evidence‐based interventions to manage infant vaccination pain (Riddell et al., 2015). The results of this study have highlighted a knowledge‐action gap that is of concern given the documented consequences of untreated pain in infants during vaccination. In order to increase efforts to protect children from the immediate and long‐term consequences of pain, effective knowledge translation (KT) strategies must be established, targeting the individual, organization or society (Stevens et al., 2021). Some researchers in the field of childhood pain have used (KT) to provide evidence‐based interventions to health workers, including nurses, and have been shown to improve health outcomes (Krauss et al., 2008). For instance, in a systematic review of KT approaches in paediatric pain specifically targeting HCPs. Gagnon et al. (2016) have shown that didactic workshops, clinician reminders and decision support, strategies have been successful in changing the conceptual use of knowledge (i.e. knowledge, attitudes and beliefs about pain) by health professionals. Stevens et al. (2014) found that KT strategies such as continuous quality improvement, chart reminders, educational materials, and audit and feedback produce significant improvements in pain assessment and management.

As all Ghanaian infants need to be vaccinated and the consequence of unmanaged infant vaccination pain, there is an urgent need for KT efforts such as didactic workshops, reminders, audits and feedback for Ghanaian nurses to implement WHO recommendations during infant vaccination will provide better patient experience, increase vaccination compliance, help address short‐ and long‐term consequences associated with vaccine‐associated pain and build trust between nurses and mothers (Taddio, 2021).

## 6 IMPLICATION FOR CLINICAL PRACTICE

Nurses should be equipped with the knowledge, resources and support needed to assist mothers in applying WHO evidence‐based recommended strategies during infant vaccination. KT strategies such as reminders, educational sessions, educational materials, audits and feedback can be used to train nurses in the clinics that provide infant vaccine injections. These strategies aim to implement the knowledge that will help nurses avoid pain during painful events such as immunization. They should also encourage the use of recommended strategies such as breastfeeding, administration of oral sweet‐tasting solutions, proper position, caregiver presence, and distraction with adequate time and resources for effective pain management in infants during vaccination while addressing their barriers. The use of these strategies can successfully reduce vaccination‐associated pain in infants and improve vaccination compliance in the Ghanaian context.

## 7 LIMITATIONS OF THE STUDY

The study was contextual; the sites of the study were limited to the Greater Accra Region of Ghana, and therefore, findings may not reflect the views of mothers who bring their babies to be vaccinated and nurses who vaccinate infants at the CWCs in other regions, particularly the rural areas. Hence, the study findings may not be widely generalizable to other contexts in Ghana. However, transferability may be considered when the context is similar.

## 8 CONCLUSION

Vaccination is of paramount importance to prevent short‐term and long‐term consequences of disease that can last a lifetime. Infant pain is a source of concern for both the nurse vaccinating the infant and the mother watching her baby in pain. Addressing this issue can positively impact nurses' management practices and improve child and system outcomes. Nurses must be trained and supported with the necessary resources to effectively manage infant vaccine‐related pain. Breastfeeding during vaccinations should be facilitated whenever possible, sweet solutions vaccinations should be given prior to injections, and infants should be properly positioned during infant vaccinations in Ghana. There should be national guidelines focusing on the application of WHO recommendations in Ghana.

## AUTHOR CONTRIBUTIONS

This study was conceptualized by EA. The data were collected by EA and analysed by EA, TSR and BJS. The manuscript was drafted and reviewed by all the authors. All the authors read and approved the manuscript.

## FUNDING INFORMATION

This research received no specific grant from any funding agency in the public, commercial or not‐for‐profit.

## CONFLICT OF INTEREST STATEMENT

The authors have declared that no conflicts of interest exist.

## Data Availability

The data that support the finding of this study are available on request from the corresponding author. Due to privacy or ethical restrictions, the study data are not publicly available.
